# Supplementary material for: K-seq, an affordable, reliable, and open Klenow NGS-based genotyping technology
Source: Plant Methods. 2021 Mar 25;17:30. doi: 10.1186/s13007-021-00733-6 (PMC7993484; doi:10.1186/s13007-021-00733-6)
Supplement: Supplementary file 1 — Additional file 1: Sdata1. Scheme primer-explorer2 algorithm. [file 13007_2021_733_MOESM1_ESM.pdf]

Supplemental data 1. Scheme algorithm

1. Identify all k-mers present on the genome

2. Annotate the positions of k-mers

3. Filter out k-mers

    3.1. with bad dust score
    3.2. with GC_content <35% or GC_content >57%

4. Select the 1000 best k-mers (By default we sort the kmers by abundance in non repetitive regions)

5. Filter kmer that does not keep the with primer3 filtering_criteria:

    5.1. MELTING_TEMPERATURE_THRESHOLD = 0
    5.2. MAX_COMP_END = 3
    5.3. MAX_COMP_ANY = 5
    5.4. MAX_COMP_ANY_FACTOR = 0.44
    5.5. "max_diff_temp": 40

6. Filter out reverse complementary k-mers

7. Select groups of 10 kmers compatibles between them using primer3

8. For each group of 10 kmer predict the products generated by all combination of pairs within a group

    8.1. Filter by correct strandness
    8.2. Filter by minimum and maximum length

9. Generate the statistics for each k-mer combination
