# Supplementary material for: K-seq, an affordable, reliable, and open Klenow NGS-based genotyping technology
Source: Plant Methods. 2021 Mar 25;17:30. doi: 10.1186/s13007-021-00733-6 (PMC7993484; doi:10.1186/s13007-021-00733-6)
Supplement: Supplementary file 3 — Additional file 3: Sdata3. Primer sequences. [file 13007_2021_733_MOESM3_ESM.pdf]

Supplemental data 3. Primer sequences

Primers K-seq
In red k-mers sequences

Wheat

adaptF-9W	TCG TCG GCA GCG TCA GAT GTG TAT AAG AGA CAG NNN GAC GTA TCA
adaptR-3W	GTC TCG TGG GCT CGG AGA TGT GTA TAA GAG ACA GNN NTG TTC ATC C
adaptR-8W	GTC TCG TGG GCT CGG AGA TGT GTA TAA GAG ACA GNN NTT CCT CTT C

Dog

adaptF-2D	TCG TCG GCA GCG TCA GAT GTG TAT AAG AGA CAG NNN GAG AGG CAG
adaptR-6D	GTC TCG TGG GCT CGG AGA TGT GTA TAA GAG ACA GNN NAA GGG AAG A
adaptR-9D	GTC TCG TGG GCT CGG AGA TGT GTA TAA GAG ACA GNN NCT CCT TTC C

Tomato

adaptF-4T	TCG TCG GCA GCG TCA GAT GTG TAT AAG AGA CAG NNN TCA TCT TC
adaptF-5T	TCG TCG GCA GCG TCA GAT GTG TAT AAG AGA CAG NNN CAA AGA AG
adaptR-5T	GTC TCG TGG GCT CGG AGA TGT GTA TAA GAG ACA GNN NTG TTG ATG

Primers PCR

IDT-8nt-NXT_i7_1	CAAGCAGAAGACGGCATACGAGATACGATCAGGTCTCGTGGGCTC*G*G
IDT-8nt-NXT_i7_2	CAAGCAGAAGACGGCATACGAGATTCGAGAGTGTCTCGTGGGCTC*G*G
IDT-8nt-NXT_i7_3	CAAGCAGAAGACGGCATACGAGATCTAGCTCAGTCTCGTGGGCTC*G*G
IDT-8nt-NXT_i7_4	CAAGCAGAAGACGGCATACGAGATATCGTCTCGTCTCGTGGGCTC*G*G
IDT-8nt-NXT_i7_5	CAAGCAGAAGACGGCATACGAGATTCGACAAGGTCTCGTGGGCTC*G*G
IDT-8nt-NXT_i7_6	CAAGCAGAAGACGGCATACGAGATCCTTGGAAGTCTCGTGGGCTC*G*G
IDT-8nt-NXT_i7_7	CAAGCAGAAGACGGCATACGAGATATCATGCGGTCTCGTGGGCTC*G*G
IDT-8nt-NXT_i7_8	CAAGCAGAAGACGGCATACGAGATTGTTCCGTGTCTCGTGGGCTC*G*G
IDT-8nt-NXT_i7_9	CAAGCAGAAGACGGCATACGAGATATTAGCCGGTCTCGTGGGCTC*G*G
IDT-8nt-NXT_i7_10	CAAGCAGAAGACGGCATACGAGATCGATCGATGTCTCGTGGGCTC*G*G
IDT-8nt-NXT_i7_11	CAAGCAGAAGACGGCATACGAGATGATCTTGCGTCTCGTGGGCTC*G*G
IDT-8nt-NXT_i7_12	CAAGCAGAAGACGGCATACGAGATAGGATAGCGTCTCGTGGGCTC*G*G
IDT-8nt-NXT_i5_1	AATGATACGGCGACCACCGAGATCTACACATATGCGCTCGTCGGCAGCG*T*C
IDT-8nt-NXT_i5_2	AATGATACGGCGACCACCGAGATCTACACTGGTACAGTCGTCGGCAGCG*T*C
IDT-8nt-NXT_i5_3	AATGATACGGCGACCACCGAGATCTACACAACCGTTCTCGTCGGCAGCG*T*C
IDT-8nt-NXT_i5_4	AATGATACGGCGACCACCGAGATCTACACTAACCGGTTCGTCGGCAGCG*T*C
IDT-8nt-NXT_i5_5	AATGATACGGCGACCACCGAGATCTACACGAACATCGTCGTCGGCAGCG*T*C
IDT-8nt-NXT_i5_6	AATGATACGGCGACCACCGAGATCTACACCCTTGTAGTCGTCGGCAGCG*T*C
IDT-8nt-NXT_i5_7	AATGATACGGCGACCACCGAGATCTACACTCAGGCTTTCGTCGGCAGCG*T*C
IDT-8nt-NXT_i5_8	AATGATACGGCGACCACCGAGATCTACACGTTCTCGTTCGTCGGCAGCG*T*C

nucleotides labelled with * have a phosphotioate bond to prevent primer degradation by the DNA polymerase
