# Supplementary material for: K-seq, an affordable, reliable, and open Klenow NGS-based genotyping technology
Source: Plant Methods. 2021 Mar 25;17:30. doi: 10.1186/s13007-021-00733-6 (PMC7993484; doi:10.1186/s13007-021-00733-6)
Supplement: Supplementary file 4 — Additional file 4: Sdata4. Detailed K-seq protocol. [file 13007_2021_733_MOESM4_ESM.pdf]

Supplemental data 4. K-seq protocol

1- First synthesis

1 µl of each forward short-primers (5µM each)
2 µl dNTPs (25mM)
4,5 µl klenow buffer 10X
200-600 ng genomic DNA
$H_2$0 until 45 µl final volume

<u>Reaction:</u>
4 min 95ºC
5 min 37ºC—add 1 µl Klenow (5U/µl Klenow Fragment (3'→5' exo)
20 min 75ºC

2- First Exonuclease digestion

+ 2µl Exonuclease I (E. coli) 20U/µl

<u>Reaction:</u>
60 min 37ºC
20 min 80ºC

3- Second synthesis

+1µl each reverse short-primers (5µM each)
+0.5 buffer Klenow 10X
+ H20 to 4 µl final volume

<u>Reaction:</u>
4 min 95ºC
5 min 37ºC. In this step add 1 µl Klenow (5U/µl)
20 min 75ºC

4- Second Exonuclease digestion

+ 1µl Exonuclease I (E. coli) 20U/µl

<u>Reaction:</u>
60 min 37ºC
20 min 80ºC

5- PCR

15 µl reaction 4
25µl Taq NEB 2X (M0270L Taq 2X Master MixNEB)
1 µl IDT-NXT adapter i7 (2.5µM)
1 µl IDT-NXT adapter i5 (2.5µM)
8µl $H_2$0

<u>Reaction:</u>
1 min 95ºC
X cycles (usually between 10 and 15 cycles. Adjust cycle number to avoid high molecular weight band smear in agarose gel electrophoresis)
30s 95ºC
20s 62ºC
30s 68ºC
Last extension step. 5 min 68ºC
