# Supplementary material for: K-seq, an affordable, reliable, and open Klenow NGS-based genotyping technology
Source: Plant Methods. 2021 Mar 25;17:30. doi: 10.1186/s13007-021-00733-6 (PMC7993484; doi:10.1186/s13007-021-00733-6)
Supplement: Supplementary file 5 — Additional file 5: Figure S1. Example of Bionalyzer results of tomato pool sample. [file 13007_2021_733_MOESM5_ESM.pdf]

Bionalyzer tomato sample

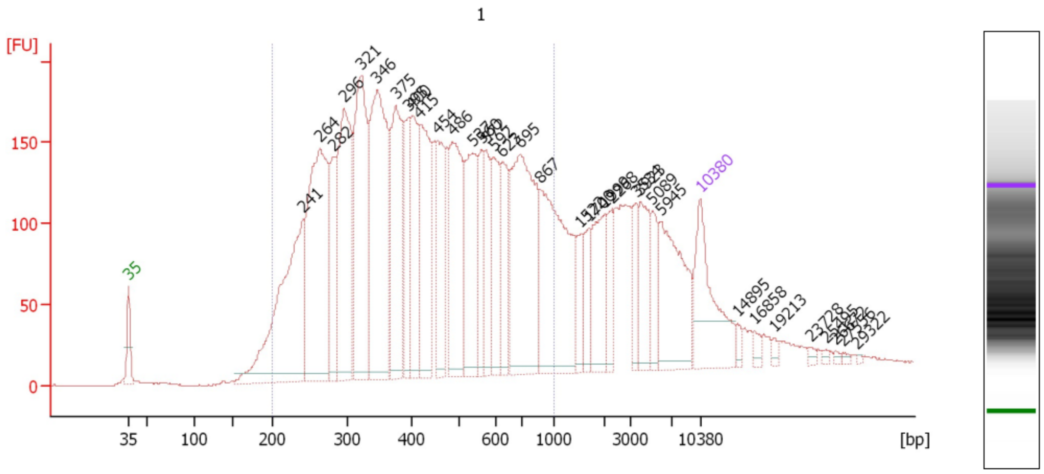

Bionalyzer size selected tomato sample

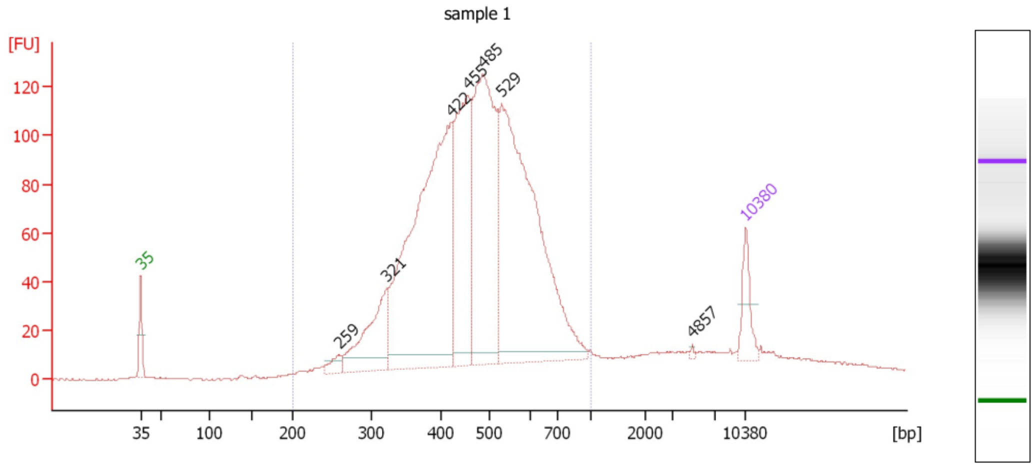

SFigure1. Bionalyzer results of tomato pool sample
