# Supplementary material for: K-seq, an affordable, reliable, and open Klenow NGS-based genotyping technology
Source: Plant Methods. 2021 Mar 25;17:30. doi: 10.1186/s13007-021-00733-6 (PMC7993484; doi:10.1186/s13007-021-00733-6)
Supplement: Supplementary file 6 — Additional file 6: Table S2. Reads and Mapping statistics of analysed samples. [file 13007_2021_733_MOESM6_ESM.pdf]

# Sup. Table 5. Reads and Maping statisitics

| | | | | | | | | | | | | | | | | |
|---|---|---|---|---|---|---|---|---|---|---|---|---|---|---|---|---|
| Tomato | | | | | | LIBRARY STATS | | | | | | | | | | |
| Reas_stats | | | | bam stats no filter | | | | | | | Bam stats >57 | | | | | |
| library | raw_reads | %raw_reads_q>30 | | reads_in_bam | mapped_reads | %mapped | properly_paired | %properly | %duplicated_reads | | reads_in_bam | mapped_reads | %mapped | properly_paired | %properly | %duplicated_reads |
| BGV006347 | 3109146 | 96.9 | | 3109146 | 3053905 | 98.2 | 2848552 | 91.6 | 0 | | 2124332 | 2124332 | 68.3% | 2082355 | 67.0% | 0 |
| BGV006777 | 9049310 | 97.5 | | 9049310 | 8946723 | 98.9 | 8545940 | 94.4 | 0 | | 6917286 | 6917286 | 76.4% | 6807863 | 75.2% | 0 |
| BGV006784 | 1395390 | 96.5 | | 1395390 | 1368657 | 98.1 | 1282412 | 91.9 | 0 | | 1012081 | 1012081 | 72.5% | 989824 | 70.9% | 0 |
| BGV006792 | 2614570 | 97.3 | | 2614570 | 2583000 | 98.8 | 2436978 | 93.2 | 0 | | 1894988 | 1894988 | 72.5% | 1861290 | 71.2% | 0 |
| BGV007145 | 2136394 | 96.9 | | 2136394 | 2101147 | 98.4 | 1964098 | 91.9 | 0 | | 1619356 | 1619356 | 75.8% | 1588883 | 74.4% | 0 |
| BGV007155 | 1725058 | 96.8 | | 1725058 | 1693701 | 98.2 | 1595958 | 92.5 | 0 | | 1313285 | 1313285 | 76.1% | 1286524 | 74.6% | 0 |
| BGV007161 | 2684288 | 96.3 | | 2684288 | 2639899 | 98.3 | 2488608 | 92.7 | 0 | | 1963881 | 1963881 | 73.2% | 1926893 | 71.8% | 0 |
| BGV007867 | 1890570 | 96.3 | | 1890570 | 1859429 | 98.4 | 1750774 | 92.6 | 0 | | 1485239 | 1485239 | 78.6% | 1450157 | 76.7% | 0 |
| BGV007869 | 3762642 | 97.1 | | 3762642 | 3723856 | 99 | 3527674 | 93.8 | 0 | | 2855539 | 2855539 | 75.9% | 2796573 | 74.3% | 0 |
| BGV007900 | 3400540 | 96.6 | | 3400540 | 3360606 | 98.8 | 3181976 | 93.6 | 0 | | 2604695 | 2604695 | 76.6% | 2552156 | 75.1% | 0 |
| Heinz1706-2 | 1837322 | 96.6 | | 1837322 | 1800028 | 98 | 1701162 | 92.6 | 0 | | 1379463 | 1379463 | 75.1% | 1342483 | 73.1% | 0 |
| Heinz1706 | 2235912 | 97.1 | | 2235912 | 2212312 | 98.9 | 2099146 | 93.9 | 0 | | 1749362 | 1749362 | 78.2% | 1713238 | 76.6% | 0 |
| HEINZ+LA1589-2 | 4122630 | 97.5 | | 4122630 | 4063211 | 98.6 | 3820806 | 92.7 | 0 | | 2929913 | 2929913 | 71.1% | 2872473 | 69.7% | 0 |
| HEINZ+LA1589 | 2292108 | 96.9 | | 2292108 | 2258330 | 98.5 | 2112176 | 92.1 | 0 | | 1658706 | 1658706 | 72.4% | 1623330 | 70.8% | 0 |
| LA1589-2 | 1688210 | 96.6 | | 1688210 | 1650068 | 97.7 | 1539184 | 91.2 | 0 | | 1132858 | 1132858 | 67.1% | 1105965 | 65.5% | 0 |
| LA1589 | 2597392 | 96.1 | | 2597392 | 2548188 | 98.1 | 2364862 | 91 | 0 | | 1782020 | 1782020 | 68.6% | 1743175 | 67.1% | 0 |
| LA2263 | 3207116 | 96.5 | | 3207116 | 3163072 | 98.6 | 2932696 | 91.4 | 0 | | 2344777 | 2344777 | 73.1% | 2297594 | 71.6% | 0 |
| LA2312 | 3236352 | 97.2 | | 3236352 | 3201903 | 98.9 | 3049030 | 94.2 | 0 | | 2525399 | 2525399 | 78.0% | 2481945 | 76.7% | 0 |
| LA2843 | 5754286 | 97.3 | | 5754286 | 5694567 | 99 | 5404532 | 93.9 | 0 | | 4385126 | 4385126 | 76.2% | 4310240 | 74.9% | 0 |
| | | | | | | | | | | | | | | | | |
| Wheat | | | | | | LIBRARY STATS | | | | | | | | | | |
| Reas_stats | | | | bam stats no filter | | | | | | | Bam stats >57 | | | | | |
| sample | raw_reads | %raw_reads_q>30 | | reads_in_bam | mapped_reads | %mapped | properly_paired | %properly | %duplicated_reads | | reads_in_bam | mapped_reads | %mapped | properly_paired | %properly | %duplicated_reads |
| 5 | 10797584 | 97.1 | | 10786324 | 10522136 | 97.6 | 8778326 | 81.4 | 0.0 | | 756239 | 756239 | 7.0 | 720872 | 44018 | 0.0 |
| AGL-001 | 3895132 | 97.1 | | 3891220 | 3835473 | 98.6 | 3638440 | 93.5 | 0.0 | | 1165378 | 1165378 | 44103 | 1148901 | 43980 | 0.0 |
| AGL-022 | 2181276 | 97.2 | | 2179264 | 2144803 | 98.4 | 2052446 | 94.2 | 0.0 | | 711571 | 711571 | 32.7 | 702280 | 32.2 | 0.0 |
| AGL-601 | 1804646 | 97.1 | | 1802874 | 1778729 | 98.7 | 1727924 | 95.8 | 0.0 | | 699750 | 699750 | 38.8 | 692810 | 38.4 | 0.0 |
| AGL-635 | 822692 | 96.6 | | 821740 | 798957 | 97.2 | 759284 | 92.4 | 0.0 | | 275216 | 275216 | 33.5 | 271974 | 33.1 | 0.0 |
| JG-1 | 11750190 | 96.9 | | 11740210 | 11562407 | 98.5 | 10848940 | 92.4 | 0.0 | | 2003827 | 2003827 | 43847 | 1963156 | 44028 | 0.0 |
| JG-6 | 9634370 | 97.0 | | 9624976 | 9485547 | 98.6 | 9011216 | 93.6 | 0.0 | | 1922227 | 1922227 | 20.0 | 1889794 | 44001 | 0.0 |
| JG-9 | 11351450 | 97.3 | | 11342150 | 11176413 | 98.5 | 9997664 | 88.1 | 0.0 | | 1082686 | 1082686 | 43960 | 1045798 | 43870 | 0.0 |
| M5 | 1674292 | 97.4 | | 1672136 | 1648781 | 98.6 | 1589920 | 95.1 | 0.0 | | 624742 | 624742 | 37.4 | 617895 | 37.0 | 0.0 |
| SVEVO | 8837918 | 97.2 | | 8830708 | 8730903 | 98.9 | 8469834 | 95.9 | 0.0 | | 3059690 | 3059690 | 34.6 | 3029728 | 34.3 | 0.0 |
| | | | | | | | | | | | | | | | | |
| Dog | | | | | | LIBRARY STATS | | | | | | | | | | |
| Reas_stats | | | | bam stats no filter | | | | | | | Bam stats >57 | | | | | |
| 18-1607 | 2917520 | 96.7 | | 2915624 | 2882978 | 98.8 | 2791176 | 95.7 | 0.0 | | 2700422 | 2700422 | 92.6 | 2637468 | 90.4 | 0.0 |
| 18-231 | 6006594 | 96.2 | | 6002540 | 5948047 | 99.0 | 5765142 | 96.0 | 0.0 | | 5492141 | 5492141 | 91.4 | 5382732 | 89.6 | 0.0 |
| 19-353 | 4562442 | 96.5 | | 4559602 | 4521292 | 99.1 | 4381804 | 96.0 | 0.0 | | 4170048 | 4170048 | 91.4 | 4087295 | 89.6 | 0.0 |
| 19-730 | 2006520 | 96.2 | | 2005176 | 1979984 | 98.7 | 1919324 | 95.7 | 0.0 | | 1831019 | 1831019 | 91.3 | 1794421 | 89.4 | 0.0 |
| 19-821 | 14336652 | 96.2 | | 14327874 | 14227315 | 99.2 | 13812776 | 96.3 | 0.0 | | 13151261 | 13151261 | 91.7 | 12911401 | 90.1 | 0.0 |
| 19-827 | 50522266 | 96.2 | | 50491188 | 50162687 | 99.3 | 48413780 | 95.8 | 0.0 | | 46829228 | 46829228 | 92.7 | 45708809 | 90.5 | 0.0 |
| | | | | | | | | | | | | | | | | |
| Potato | | | | | | LIBRARY STATS | | | | | | | | | | |
| Reas_stats | | | | bam stats no filter | | | | | | | Bam stats >57 | | | | | |
| Rudolph | 2771878 | 97.0 | | 2766440 | 2678469 | 96.6 | 2453710 | 88.5 | 0.0 | | 1430893 | 1430893 | 51.6 | 1393103 | 50.3 | 0.0 |
| | | | | | | | | | | | | | | | | |
| Eggplant | | | | | | LIBRARY STATS | | | | | | | | | | |
| Reas_stats | | | | bam stats no filter | | | | | | | Bam stats >57 | | | | | |
| Listada | 2334444 | 96.9 | | 2330440 | 2246368 | 96.2 | 2063334 | 88.4 | 0.0 | | 282126 | 1282126 | 54.9 | 1262873 | 54.1 | 0.0 |
| | | | | | | | | | | | | | | | | |
| Pepper | | | | | | LIBRARY STATS | | | | | | | | | | |
| Reas_stats | | | | bam stats no filter | | | | | | | Bam stats >57 | | | | | |

Sup. Table 5. Reads and Maping statisitics

| | | | | | | | | | | | | | | | | | |
|---|---|---|---|---|---|---|---|---|---|---|---|---|---|---|---|---|---|
| Dulcinea | 1355328 | 97.4 | | 1351854 | 1321858 | 97.5 | 1254046 | 92.5 | 0.0 | | | 1003401 | 1003401 | 74.0 | 989987 | 73.0 | 0.0 |
| | | | | | | | | | | | | | | | | | |
| Petunia | | | | | | LIBRARY STATS | | | | | | | | | | | |
| Reas_stats | | | | bam stats no filter | | | | | | | Bam stats >57 | | | | | | |
| Petunia-hibrida | 1683972 | 96.9 | | 1677882 | 1642380 | 97.9 | 1507054 | 89.8 | 0.0 | | 722871 | 722871 | 43.1 | 702592 | 41.9 | 0 | |
