# Supplementary figures and images for: K-seq, an affordable, reliable, and open Klenow NGS-based genotyping technology
Source: Plant Methods. 2021 Mar 25;17:30. doi: 10.1186/s13007-021-00733-6 (PMC7993484; doi:10.1186/s13007-021-00733-6)

### Tomato

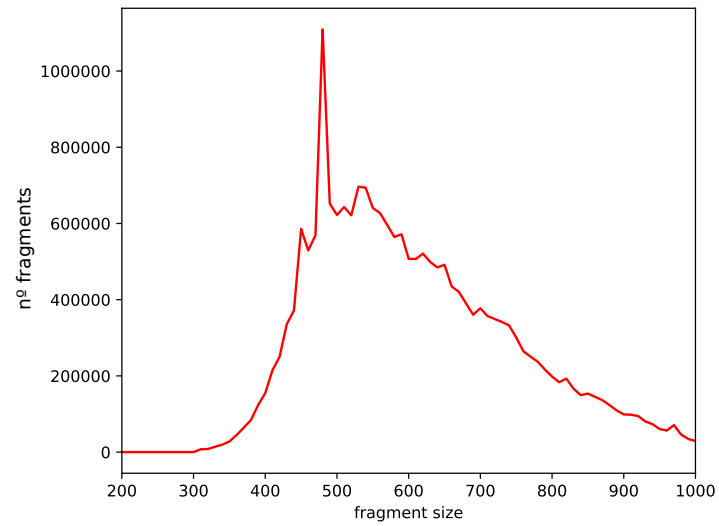

### Wheat

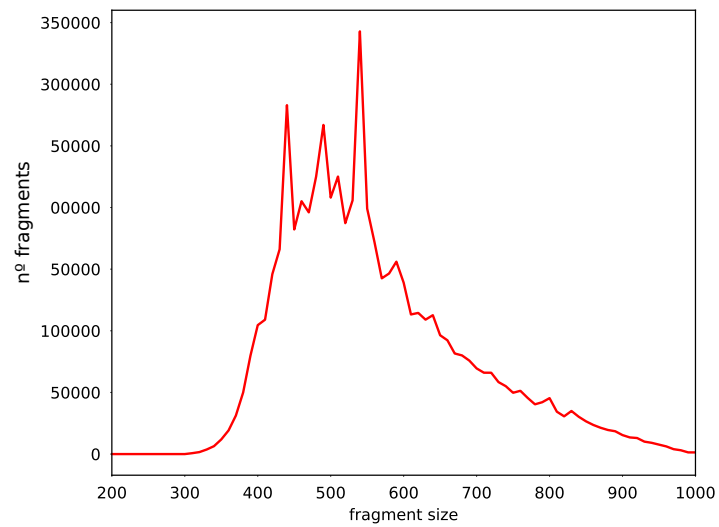

### Dog

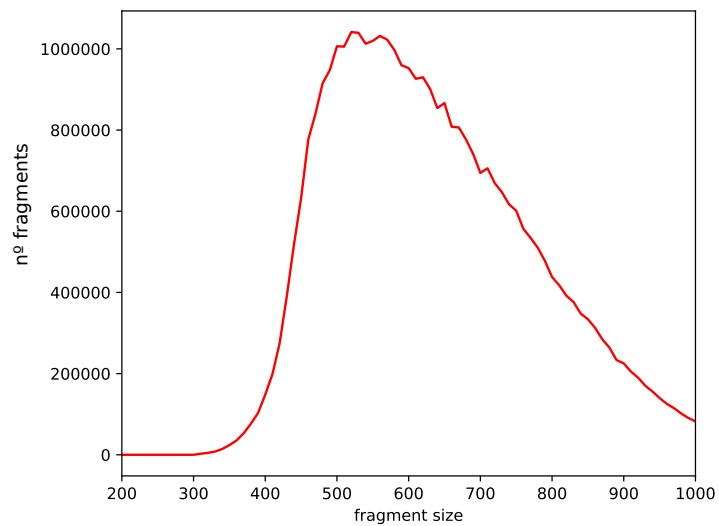

SFigure 2. Size distribution of mapped fragments

Supplement: Supplementary file 7 — Additional file 7: Figure S2. Size distribution of mapped sequenced fragments. [file 13007_2021_733_MOESM7_ESM.pdf]

Dog

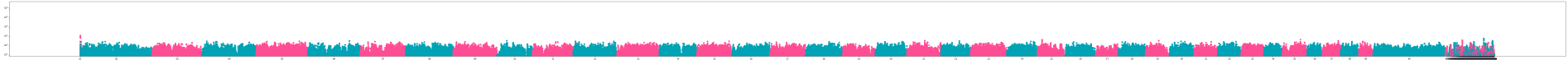

Tomato

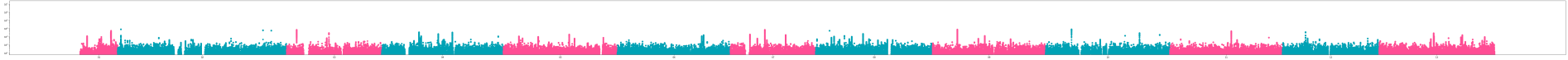

Wheat

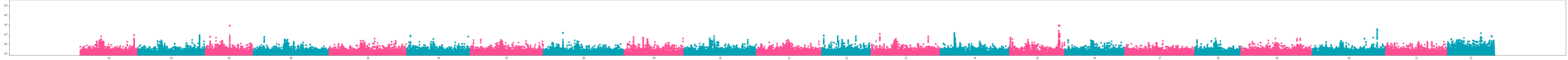

SFigure 3. Distribution of mapped fragments along genome

Supplement: Supplementary file 8 — Additional file 8: Figure S3. Distribution of mapped sequenced fragments along genomes. [file 13007_2021_733_MOESM8_ESM.pdf]

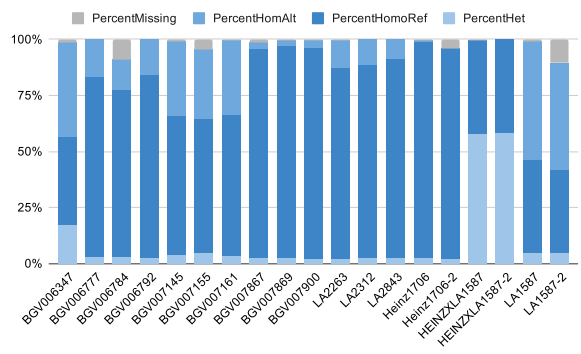

Tomato

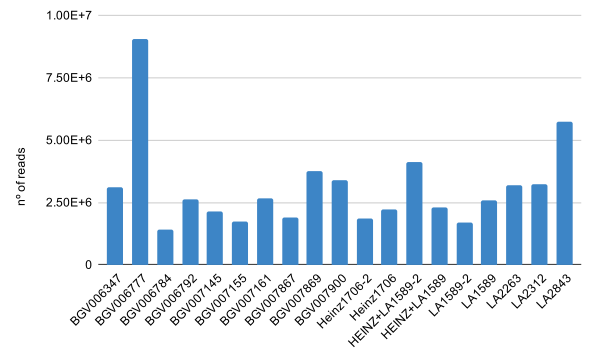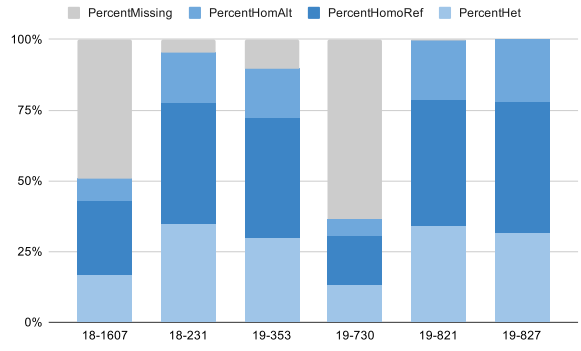

Dog

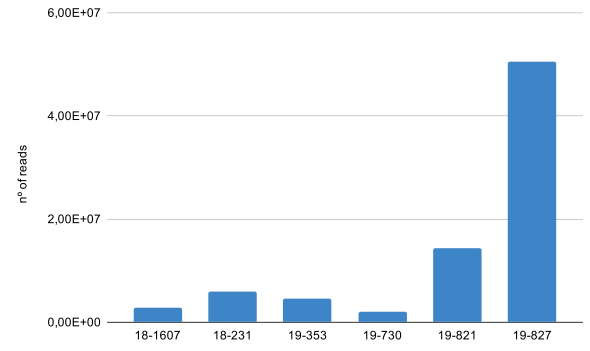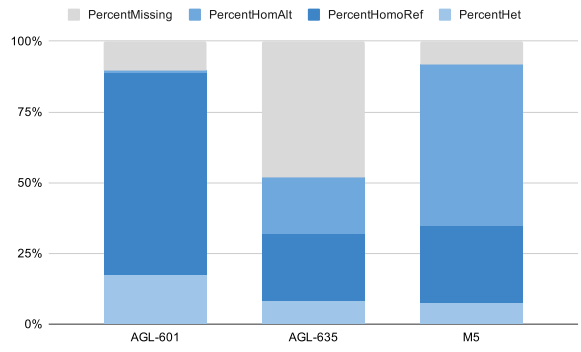

Wheat

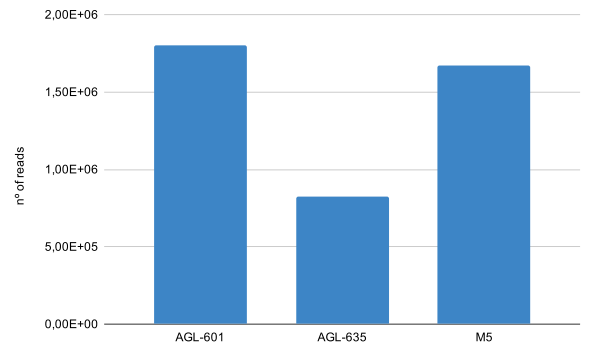

SFigure 4. SNP and mapping statistics

Supplement: Supplementary file 9 — Additional file 9: Figure S4. SNP and mapping statistics. [file 13007_2021_733_MOESM9_ESM.pdf]

A

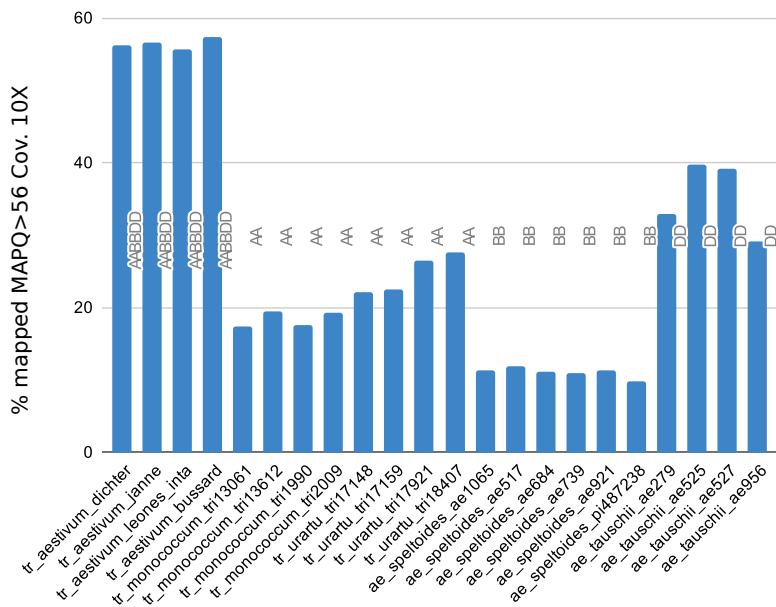

B

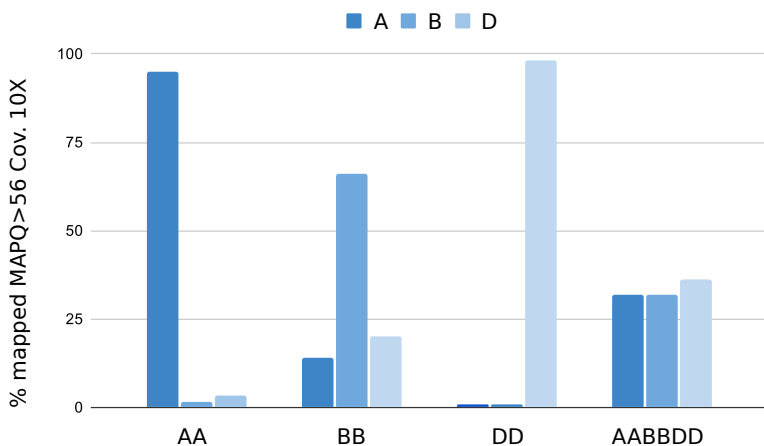

Supplement: Supplementary file 11 — Additional file 11: Figure S5. Mapping statistics of GBS wheat samples. [file 13007_2021_733_MOESM11_ESM.pdf]
