# Supplementary material for: K-seq, an affordable, reliable, and open Klenow NGS-based genotyping technology
Source: Plant Methods. 2021 Mar 25;17:30. doi: 10.1186/s13007-021-00733-6 (PMC7993484; doi:10.1186/s13007-021-00733-6)
Supplement: Supplementary file 10 — Additional file 10: Table S1. Statistics of primer-explored2 predicted regions for wheat, tomato and dog genomes. [file 13007_2021_733_MOESM10_ESM.pdf]

Sup. table 4. Statistics predicted regions

	Dog	Tomato	Wheat
Genome size	2,410,976,875	824,674,700	14,547,261,565
Prediction size	6,038,783	1,022,464	1,534,057
Coverage 10X size	19,368,633	3,708,530	920964
% genome predicted	0.25%	0.12%	0.01%
%genome coverage 10X	0.80%	0.45%	0.006%
% gene genome	50.51%	13.19%	2.33%
% gene prediction	53.0%	20.98%	0.40%
% gene coverage 10X	49.23%	28.24%	2.8%
% repetitive genome	42.83%	64.03%	43.65%
%repetitive prediction	76.4%	72.2%	23.10%
% repetitive coverage 10X	55.33%	62.71%	22.56%
% prediction coverage 10X	3.60%	4.06%	2.28%
