# Supplementary material for: K-seq, an affordable, reliable, and open Klenow NGS-based genotyping technology
Source: Plant Methods. 2021 Mar 25;17:30. doi: 10.1186/s13007-021-00733-6 (PMC7993484; doi:10.1186/s13007-021-00733-6)
Supplement: Supplementary file 12 — Additional file 12: Table S6. Comparation of statistic from GBS and K-seq of wheat samples. [file 13007_2021_733_MOESM12_ESM.pdf]

	Wheat 6N K-seq	Wheat 6N GBS
**Samples**	3	4
**Reads**	0.8-1.8 millions	1.6-2 millions
**% sequence quality>30 average**	96.87	88.4
**% mapping properly average**	94.43	97.9
**% mapping properly q>57 average**	36.16	56.2
**nºSNPs**	5047	16350
**% call rate**	78	85.3
**Filtered SNPs (60% call rate, >10x)**	655	180
**Filtered SNPs % call rate**	77.7	80
**GBS samples:**		
**Hexaploid wheat samples**	Keilwagen J,et al. Detecting Large Chromosomal Modifications Using Short Read Data From Genotyping-by-Sequencing. Frontiers in Plant Science. 2019;10:1133	
Ditcher		
Leones_INTA		
Janne		
Bussard		
**Diploid Wheat samples**	Bernhardt N, Brassac J. Genomic and phylogenetic data for the identification of ancient and recurrent hybridizations in wheat taxa [Internet]. e!DAL - Plant Genomics and Phenomics Research Data Repository (PGP), IPK Gatersleben, Seeland OT Gatersleben, Corrensstraße 3, 06466, Germany; 2019. Disponible en: https://doi.ipk-gatersleben.de:443/DOI/005cf59f-a628-416d-a0d5-69dd708201c3/757b83b3-dc23-42fd-a[illegible]	
ae_tauschii_ae956		
tr_urartu_tri17921		
tr_monococcum_tri1990		
tr_urartu_tri18407		
tr_monococcum_tri2009		
ae_speltoides_ae921		
ae_speltoides_ae517		
tr_urartu_tri17148		
ae_tauschii_ae525		
tr_urartu_tri17159		
tr_monococcum_tri13061		
tr_monococcum_tri13612		
ae_speltoides_ae684		
ae_tauschii_ae279		
ae_speltoides_ae739		
ae_tauschii_ae527		
ae_speltoides_ae1065		
ae_speltoides_pi487238		
