# Supplementary material for: K-seq, an affordable, reliable, and open Klenow NGS-based genotyping technology
Source: Plant Methods. 2021 Mar 25;17:30. doi: 10.1186/s13007-021-00733-6 (PMC7993484; doi:10.1186/s13007-021-00733-6)
Supplement: Supplementary file 13 — Additional file 13: Table S3. Data of samples used in K-seq analysis. [file 13007_2021_733_MOESM13_ESM.pdf]

Sup. Table 1 K-seq samples data

Sample	Variety/code	Species	Genome	Ploidy	Sex	Genome sequence
AGL-601	CHINESE SPRING	Triticum aestivum subsp vulgare	AABBDD	6N		161010_Chinese_Spring_v1.0_pseudomolecules
AGL-635	THATCHER	Triticum aestivum subsp vulgare	AABBDD	6N		161010_Chinese_Spring_v1.0_pseudomolecules
M5	BGE18908	Triticum aestivum subsp speltoic	AABBDD	6N		161010_Chinese_Spring_v1.0_pseudomolecules
AGL-001	BGE047503	Triticum turgidum subsp dicocco	AABB	4N		161010_Chinese_Spring_v1.0_pseudomolecules
AGL-022	BGE047513	Triticum turgidum subsp turgidur	AABB	4N		161010_Chinese_Spring_v1.0_pseudomolecules
SVEVO	SVEVO	Triticum turgidum subsp durum	AABB	4N		161010_Chinese_Spring_v1.0_pseudomolecules
JG-1	Seed give F. Vazque	Triticum urartu	AA	2N		161010_Chinese_Spring_v1.0_pseudomolecules
JG-9	Seed give F. Vazque	Triticum monococum subsp boe	AA	2N		161010_Chinese_Spring_v1.0_pseudomolecules
5	Seed give F. Vazque	Aegilops speltoides	BB	2N		161010_Chinese_Spring_v1.0_pseudomolecules
JG-6	Seed give F. Vazque	Aegilops tauschii	DD	2N		161010_Chinese_Spring_v1.0_pseudomolecules
Dulcinea	Dulcinea	Capsicum Annum		2N		CA_000512255.2_ASM51225v2_genomic
Listada de gandia	Listada de gandia	Solanum melogena		2N		Eggplant_V3_Chromosomes
Rudolph	Rudolph	Solanum tubersoum		4N		GCF_000226075.1_SolTub_3.0_genomic
Petuniaxhibrida		Petuniaxhibrida		2N		Petunia_axillaris_v1.6.2_genome_HiC
Heinz1706	Heinz1706	Solanum lycopersicum		2N		S_lycopersicum_chromosomes.2.50
LA1589	LA1589	Solanum pimpinelifollium		2N		S_lycopersicum_chromosomes.2.50
BGV006777	BGV006777	Solanum lycopersicum var. cerasiforme		2N		S_lycopersicum_chromosomes.2.50
BGV006784	BGV006784	Solanum lycopersicum var. cerasiforme		2N		S_lycopersicum_chromosomes.2.50
BGV006792	BGV006792	Solanum lycopersicum var. cerasiforme		2N		S_lycopersicum_chromosomes.2.50
LA2263	LA2263	Solanum lycopersicum var. cerasiforme		2N		S_lycopersicum_chromosomes.2.50
LA2312	LA2312	Solanum lycopersicum var. cerasiforme		2N		S_lycopersicum_chromosomes.2.50
LA2843	LA2843	Solanum lycopersicum var. cerasiforme		2N		S_lycopersicum_chromosomes.2.50
BGV007867	BGV007867	Solanum lycopersicum var. cerasiforme		2N		S_lycopersicum_chromosomes.2.50
BGV007869	BGV007869	Solanum lycopersicum var. cerasiforme		2N		S_lycopersicum_chromosomes.2.50
BGV007900	BGV007900	Solanum lycopersicum var. cerasiforme		2N		S_lycopersicum_chromosomes.2.50
BGV007145	BGV007145	Solanum pimpinelifollium		2N		S_lycopersicum_chromosomes.2.50
BGV007155	BGV007155	Solanum pimpinelifollium		2N		S_lycopersicum_chromosomes.2.50
BGV007161	BGV007161	Solanum pimpinelifollium		2N		S_lycopersicum_chromosomes.2.50
BGV006347	BGV006347	Solanum pimpinelifollium		2N		S_lycopersicum_chromosomes.2.50
18/231	Poodle	Cannis lupus familares		2N	Female	GCF_000002285.3_CanFam3.1_genomic
19/827	Spaniel breton	Cannis lupus familares		2N	Male	GCF_000002285.3_CanFam3.1_genomic
19/353	Yorkshire terrier	Cannis lupus familares		2N	Female	GCF_000002285.3_CanFam3.1_genomic
19/730	mix	Cannis lupus familares		2N	Female	GCF_000002285.3_CanFam3.1_genomic
19/821	mix	Cannis lupus familares		2N	Male	GCF_000002285.3_CanFam3.1_genomic
18/1607	mix	Cannis lupus familares		2N	Male	GCF_000002285.3_CanFam3.1_genomic
