# Supplementary material for: K-seq, an affordable, reliable, and open Klenow NGS-based genotyping technology
Source: Plant Methods. 2021 Mar 25;17:30. doi: 10.1186/s13007-021-00733-6 (PMC7993484; doi:10.1186/s13007-021-00733-6)
Supplement: Supplementary file 14 — Additional file 14: Table S4. Breed and genomic information for WGS dog samples. [file 13007_2021_733_MOESM14_ESM.pdf]

Name_ID_Study	Name_ID_SRA	Breed/CommonNa	BioProject	BioSample	SRA	Sex	
Alaskan Malamute	AlaskanMalamute(	Alaskan Malamute	PRJNA448733	SAMN08872810	SRR7107992	F	
American Cocker S	CockerSpanielAme	Cocker Spaniel (An	SRP108905	SRR5664964	SRR5664964	M	
Beagle 1	CFA.117995	Beagle	PRJNA263947	SAMN06159681	SRR7107976	M	
Bearded Collie 2	BeardedCollie02	Bearded Collie	PRJEB16012	SAMEA4505492	SRR7107609	M	
Belgian Malinois 4	MA0163	Belgian Malinois	PRJEB16012	SAMEA104032048	SRR7107526	F	
Black and Tan Coo	Coonhound01	Black and Tan Coo	PRJNA263947	SAMN04196853	SRR7107925	F	
Boston Terrier	BostonTerrier01	Boston Terrier	PRJNA448733	SAMN08872906	SRR7120145	F	
Boxer	Boxer01	Boxer	PRJNA255370	SAMN02921305	SRR7107773	F	
Bull Terrier 1	BT1021	Bull Terrier	PRJEB16012	SAMEA104125116	SRR7107556	F	
Bull Terrier Miniat	BT007	Miniature Bull Terı	PRJEB16012	SAMEA4506897	SRR7107631	M	
Bulldog	Bulldog01	Bulldog	PRJNA288568	SAMN03801654	SRR2095477	F	
Chihuahua 1	Chihuahua01	Chihuahua	PRJNA288568	SAMN03801656	SRR2095478	F	
Chinese Crested 1	ChineseCrested01	Chinese Crested	PRJNA261736	SAMN03075611	SRR7107801	M	
Chow Chow 2	ChowChow02	Chow Chow	PRJNA288568	SAMN03801658	SRR2094392	F	
Coyote 1	Coyote01	Coyote	PRJNA255370	SAMN02921301	SRR7107770		
Dachshund 1	CFA.107835	Dachshund	PRJNA263947	SAMN06159670	SRR7107965	M	
Dalmatian 2	Dalmatian01	Dalmatian	PRJNA448733	SAMN08872976	SRR7120157	F	
Doberman Pinsche	DO242	Doberman Pinsche	PRJEB16012	SAMEA4505489	SRR7107606	F	
English Cocker Spa	CP003	English Cocker Spa	PRJEB16012	SAMEA4506900	SRR7107632	M	
English Pointer 1	EnglishPointer01	Pointer (English)	PRJNA288568	SAMN03801665	SRR2094398	M	
English Springer Sp	EnglishSpringerSpa	English Springer Sp	PRJNA263947	SAMN03580391	SRR7107883	M	
German Shepherd	DS043	German Shepherd	PRJEB16012	SAMEA4506895	SRR7107629	F	
German Wirehaire	GermanWirehaire(	German Wirehaire	PRJEB13468	SAMEA3928144	SRR7107585	F	
Golden Retriever 1	140447_S11	Golden Retriever	PRJNA448733	SAMN08873033	SRR7120160	M	
Great Dane 1	DD116	Great Dane	PRJEB16012	SAMEA104091557	SRR7107534	M	
Grey Wolf (Canis L	WolfTibetan01	Grey Wolf (Canis L	PRJNA448733	SAMN03652997	SRR7107906	M	
Grey Wolf 10	Wolf19	Grey Wolf	PRJNA255370	SAMN02921311	SRR7107777	M	
Greyhound 2	Greyhound02	Greyhound	PRJNA247491	SAMN03067872	SRR7107789	M	
Grossspitz	GS104	Grossspitz	PRJEB16012	SAMEA104105252	SRR7107551	F	
Iberian Wolf (Canis	Wolf39	Iberian Wolf (Canis	PRJNA318403	SAMN04851099	SRR7107942	F	
Irish Setter	IrishSetter01	Irish Setter	PRJNA448733	SAMN08873112	SRR7120167	F	
Irish Water Spanie	IrishWaterSpaniel(	Irish Water Spanie	PRJNA448733	SAMN08873115	SRR7120168	F	
Italian Greyhound	ItalianGreyhound0	Italian Greyhound	PRJNA288568	SAMN03801673	SRR2094401	M	
Jack Russell Terrier	JackRussellTerrier(	Jack Russell Terrier	PRJNA263947	SAMN03580384	SRR7107878	M	
Jindo	Jindo01	Jindo	PRJDB2266	SAMD00009664	SRR7107521	M	
Labrador Retriever	91317_S7	Labrador Retriever	PRJNA448733	SAMN08873148	SRR7120181	M	
Miniature Poodle 2	MiniaturePoodle01	Miniature Poodle	PRJNA448733	SAMN08873175	SRR7120187	F	
MIX: Dachsund	CFA.118000	MIX: Dachsund	PRJNA263947	SAMN06159686	SRR7107981	M	
MIX: Golden Retrie	GR1078	MIX: Golden Retrie	PRJEB16012	SAMEA104091572	SRR7107549	F	
MIX: Kerry Blue Te	MIX_KerryBlueTer	MIX: Kerry Blue Te	PRJNA263947	SAMN04196846	SRR7107918	M	
MIX: Labrador Retı	LA2382	MIX: Labrador Retı	PRJEB16012	SAMEA104125075	SRR7107552	F	
MIX: Mixed Breed	MI016	MIX: Mixed Breed	PRJEB16012	SAMEA4506893	SRR7107627	F	
MIX: Siberian Husk	MixedBreed07	MIX: Siberian Husk	PRJNA448733	SAMN08873188	SRR7120190	F	
Pekingese	Pekingese01	Pekingese	PRJNA288568	SAMN03801676	SRR2095500	F	
Pomeranian	Pomeranian01	Pomeranian	PRJEB16012	SAMEA4506892	SRR7107626	M	
Portuguese Poden	PortuguesePodeng	Portuguese Poden	PRJNA263947	SAMN03580388	SRR7107881	M	
Portuguese Water	PortugueseWaterD	Portuguese Water	PRJNA448733	SAMN08873227	SRR7120201	M	
Red Wolf 1	Wolf25	Red Wolf	PRJNA255370	SAMN02921317	SRR7107783	F	
Rottweiler 1	165414_S20	Rottweiler	PRJNA448733	SAMN08873243	SRR7120204	F	
Saint Bernard 1	SaintBernard01	Saint Bernard	PRJNA288568	SAMN03801685	SRR2095502	F	
Saluki 1	Saluki01	Saluki	PRJNA288568	SAMN03801686	SRR2095503	F	
Samoyed 1	Samoyed01	Samoyed	PRJNA448733	SAMN08873258	SRR7120211	F	
Scottish Terrier 1	ScottishTerrier01	Scottish Terrier	PRJNA288568	SAMN03801688	SRR2094409	F	
Shiba Inu 1	CFA.107839	Shiba Inu	PRJNA263947	SAMN05770194	SRR7107955	F	
Siberian Husky 1	SiberianHusky01	Siberian Husky	PRJNA288568	SAMN03801690	SRR2095539	F	
Sloughi 1	Sloughi02	Sloughi	PRJEB16012	SAMEA4506885	SRR7107619	M	

Spinone Italiano	CFA.109669	Spinone Italiano	PRJNA263947	SAMN06159677	SRR7107972	F	
Standard Poodle 1	CFA.107842	Standard Poodle	PRJNA263947	SAMN06159675	SRR7107970	F	
Standard Schnauze	CFA.118001	Standard Schnauze	PRJNA263947	SAMN06159687	SRR7107982	M	
Tibetan Terrier 2	TibetanTerrier02	Tibetan Terrier	PRJNA263947	SAMN03580406	SRR7107898	M	
Toy Poodle	ToyPoodle01	Toy Poodle	PRJNA288568	SAMN03801692	SRR2095540	F	
West Highland Wh	WW558	West Highland Wh	PRJEB16012	SAMEA104091561	SRR7107538	F	
Yorkshire Terrier 2	PER00075	Yorkshire Terrier	PRJNA448733	SAMN08873470	SRR7120258	F	
Yorkshire Terrier 3	BAN00032	Yorkshire Terrier	PRJNA448733	SAMN08873449	SRR7120237	F	
Yorkshire Terrier 3	PER00204	Yorkshire Terrier	PRJNA448733	SAMN08873476	SRR7120264	F	
Yorkshire Terrier 4	BAN00041	Yorkshire Terrier	PRJNA448733	SAMN08873450	SRR7120238	M	
Yorkshire Terrier 4	PER00409	Yorkshire Terrier	PRJNA448733	SAMN08873490	SRR7120278	F	
Standard Poodle 2	StandardPoodle01	Standard Poodle	PRJNA288568	SAMN03801691	SRR2095325	F	
Brittany	BrittanySpaniel01	Brittany	PRJNA288568	SAMN03801653	SRR2094390	M	
