# Supplementary material for: K-seq, an affordable, reliable, and open Klenow NGS-based genotyping technology
Source: Plant Methods. 2021 Mar 25;17:30. doi: 10.1186/s13007-021-00733-6 (PMC7993484; doi:10.1186/s13007-021-00733-6)
Supplement: Supplementary file 15 — Additional file 15: Table S5. Data of tomato GBS samples. [file 13007_2021_733_MOESM15_ESM.pdf]

Sup. table 3GBS tomate samples data

SRA Run	bioproject	biosample	Accession Number	Species	Country		
SRR12171193	PRJNA644494	SAMN09229607	BGV006347-GBS	Solanum pimpine	Peru: Morropon: La Matanza		
SRR12171275	PRJNA644494	SAMN09229618	BGV006777-GBS	Solanum lycoper	Ecuador: Tena: Puerto Misahualli		
SRR12171253	PRJNA644494	SAMN15045111	BGV006784-GBS	Solanum lycopersicu	Ecuador		
SRR12171238	PRJNA644494	SAMN09229620	BGV006792-GBS	Solanum lycoper	Ecuador: Tena: Puerto Napo		
SRR12171242	PRJNA644494	SAMN15045119	BGV007145-GBS	Solanum pimpinellifol	Unknown		
SRR12171271	PRJNA644494	SAMN09229648	BGV007161-GBS	Solanum pimpine	Ecuador: Pedernales: Pedernales		
SRR12171235	PRJNA644494	SAMN09229662	BGV007867-GBS	Solanum lycopersicu	Mexico: Uman: Uman		
SRR12171232	PRJNA644494	SAMN15045109	BGV007869-GBS	Solanum lycoper	Mexico		
SRR12171234	PRJNA644494	SAMN09229672	BGV007900-GBS	Solanum lycoper	Mexico: Huauchinango: Huauchinango		
SRR12171265	PRJNA644494	SAMN15045106	HEINZ1706-1-GE	Solanum lycoper	USA		
SRR12171264	PRJNA644494	SAMN15045106	HEINZ1706-2-GE	Solanum lycoper	USA		
SRR12171212	PRJNA644494	SAMN15472363	HEINZ1706+LA1	Solanum	Unknown		
SRR12171303	PRJNA644494	SAMN15472363	HEINZ1706+LA1	Solanum	Unknown		
SRR12181073	PRJNA644494	SAMN15488734	LA1589-1-GBS	Solanum pimpine	Peru		
SRR12181072	PRJNA644494	SAMN15488734	LA1589-1-GBS	Solanum pimpine	Peru		
SRR12182112	PRJNA644494	SAMN15045108	BGV007155-GBS	Solanum pimpine	Ecuador		
SRR12182111	PRJNA644494	SAMN15045107	LA2312-GBS	Solanum lycoper	Peru		
SRR12182110	PRJNA644494	SAMN15045118	LA2843-GBS	Solanum lycoper	Peru		
SRR12182109	PRJNA644494	SAMN15045114	LA2263-GBS	Solanum lycoper	Peru		
